# Utilizing Industrial Sludge Ash in Brick Manufacturing and Quality Improvement

**DOI:** 10.3390/ma17112568

**Published:** 2024-05-27

**Authors:** Yu-Ming Huang, Chao-Shi Chen, Chen-Chung Chen, Jian-Wen Lai

**Affiliations:** Department of Resources Engineering, National Cheng Kung University, Tainan City 701, Taiwanianlai4568@gmail.com (J.-W.L.)

**Keywords:** carbonation, industrial sludge ash, engineering properties, mechanical properties

## Abstract

This research demonstrates changes in the behaviors and characteristics of sintered bricks while using industrial sludge ash (ISA) and waste glass (WG) as a replacement for clay in the brick manufacturing procedure. Owing to the limited amount of available land in Taiwan, it is becoming increasingly difficult to locate suitable sites for sanitary landfills, which is a common final disposal method for ash that is produced during thermal treatment in sludge factories. To meet the urgent need for land, the final waste disposal must convert this waste into a new resource. This research investigated the feasibility of using general industrial sludge ash waste, due to its abundance and high potential as a raw material in producing bricks. The result of this study shows that the bricks made from ISA and WG under a certain mixture proportion (ISA50%/WG40%/Clay10%) had excellent industrial potentials, such as compressive strength and water absorption rate. However, owing to the wide variety of components from different sources of ISA, the mixture proportion might vary accordingly. This study also analyzed the incineration index, proportion design, and process improvement, as well as investigating the possibility of increasing the total use of sludge ash as a resource. This study shows the potentials of utilizing wastes as raw materials in industrial manufacturing procedures. Therefore, more wastes can be tested and turned into resources in the future.

## 1. Introduction

As the amount of industrial sludge waste has increased rapidly over recent decades and such waste comprises various components, thermal treatment has become increasingly necessary. Owing to the improvements in both technology and equipment, thermal treatment is now indispensable for managing sludge waste [1]. Landfills represent the final disposal method for industrial sludge ash (ISA), which is produced during the process of thermal treatment in sludge treatment plants. However, issues that inevitably arise include the high cost of suitable sites and the potential pollution produced during shipping procedures or the landfill itself. These can be mitigated via resourcification of the waste.

An adobe brick that is made out of clay has a mass of about 2.2 to 2.5 kg, which drops to 1.7 to 1.8 kg after sintering. It takes 36 tons of clay to make 10,000 bricks. About 340 billion tons of clay are mined annually to produce building bricks globally [2,3].

The main sources of clay in Taiwan are mudstone and shale. In compliance with government policy and the need to lower costs, the current additives are building residues, reservoir and river sediment, ash, and rice husks. However, the yield rate decreases due to the variety of these sources and the problems caused by water content differences in the manufacturing processes [4].

Much research about utilizing different kinds of waste for making bricks has been published over recent years, which include leather sludge [5], water treatment plant sludge [6], sewage sludge [7], and reservoir sludge [8]. These contain common earth elements such as Ca, Si, and Al, and other inorganic compounds, which leads to a high potential for their reuse as materials [9].

Research has shown that clay could be partially replaced by various kinds of sludge in brick manufacturing and still meet the building criteria in both strength and water absorption rate. However, such research has only focused on the properties of a single type of waste, which does not suit the complexity of industrial sludge. In particular, every batch of sludge might vary due to the sources of sludge treatment received at a manufacturing plant. Therefore, the amount of substitute sludge cannot be the only factor in effectively utilizing industrial sludge as brick material. Extra additives that can modify the properties must be considered to unify the brick quality.

Glass, as flux during sintering, decreases the melting point and enhances the quality of the bricks [10,11,12]. Adding glass, rice husk, ash, and marble sludge and replacing 25% of clay with glass increases the compressive strength by 40% [13], and this occurs when adding 5~30% of glass into clay samples that contain small amounts of the Zn-plating sludge. As the amount of glass that is added to the specimen increases, the compressive strength increases and the water absorption rate decreases.

The increase in brick quality is mainly caused by melting of the glass powder, which forms a dense crystal layer on the surface of the specimen when the sintering temperature reaches 800~900 °C, regardless of the sintering time. This layer prevents liquids from being absorbed by the specimen and, therefore, increases the compressive strength of the specimen [14,15].

There is growing demand for large televisions, mobile devices, and monitors in cars (LCD). Taiwan, as a major LCD manufacturing country, holds 11% of the global exporting market share [16]. It produces 8000 tons of waste glass (WG) every year, including 10% of scrape products and 90% of defective products. In addition to being used as recycled building materials after pulverization, landfills are a common final disposal method.

This places a heavy burden on the environment, similar to industrial sludge. This study investigates the feasibility of using industrial sludge ash as a substitute for clay in making bricks and evaluates the effect of using waste glass as melting flux on brick quality, aiming to increase the utilization ratio of industrial sludge ash.

## 2. Materials and Methods

### 2.1. Characterization and Conditioning of Raw Materials

In this research, we used ISA that is the product of thermal treatment (above 800 °C) in a sludge treatment facility. The categories of sludge that are approved for processing include organic sludge (D-0901), inorganic sludge (D-0902), and a sludge mixture (D-0999). The sources of this sludge are from the wastewater treatment industry, dyeing and coating manufacturing industry, microchip manufacturing industry, electronic component manufacturing industry, and other chemical manufacturing industries.

The raw material we used was clay bought from a traditional brick factory, and this clay was crushed by a wheel press with its impurities removed. The processed clay was then dried in an oven (105 °C) after basic characteristic analysis. After drying, primary screening (for removal of impurities), crushing, grinding, and sieving (#100), we obtained the dry clay powder for later use. To prevent it from becoming wet, we preserved the clay powder in a sealed plastic bag and dried it in an oven at 105 °C before use. The source of the waste glass (WG) was glass powder from a glass recycling facility. This glass powder was obtained by crushing recycled LCD panels, and the resulting colorless, clear glass powder was then dried and sieved (#100) before being placed in a sealed plastic bag for later use.

### 2.2. Preparation of Bricks

Before making adobe brick, the materials were dried, ground, and sieved according to the experimental requirements. This study prepared three pieces of bricks for each mixture proportions. The quantities of materials for making a set of three blocks were calculated. Each 5 cm^3^ block took about 250 g of the dried material. The volumes of added water relative to the particle size were 20% and 30% of the weight of the specimen. We poured water into a blender before the other material in case the powder became stuck together and made it more difficult to blend. To increase their viscosity, the blended specimens were left to mature for 24 h.

**Figure 1:**
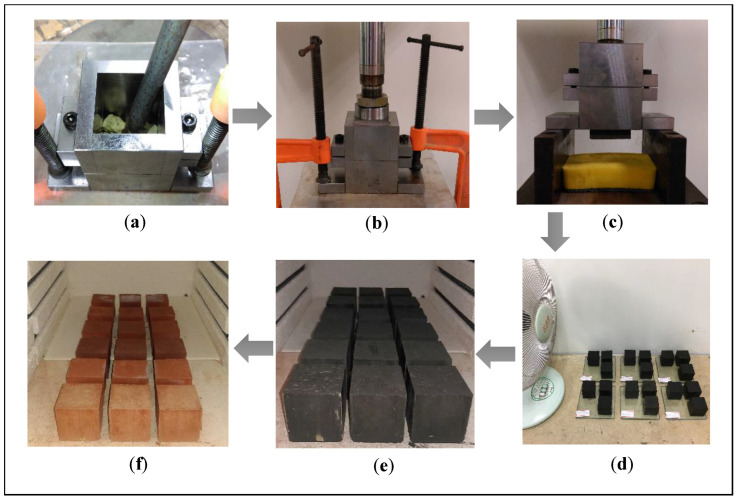
The process of brick manufacturing: (**a**) compacting, (**b**) molding, (**c**) demolding, (**d**) drying, (**e**) firing, and (**f**) final product.

As shown in Figure 1, the adobe bricks were compacted to expel the air and increase their density. The specimen was then formed by compression under a pressure of 4 MPa within the mold. The extra material outside the mold was trimmed. We obtained the adobe brick by removing the mold from the compression molding machine and the mold itself. Fewer cracks on the surface of the brick developed if the drying time was prolonged. The air-drying time was set to 48 h in this study. Afterward, the brick was placed into a combustion chamber but only after removing most of the water within the brick. However, it must not be completely dry. Thus, the brick was dried in the oven at 70 °C for 24 h before being placed in the combustion chamber. The sintering parameters in this research refer to common bricks manufacturing procedure from local brick factories. A 36 °C/h rate of temperature change was applied to reach the sintering temperature, which was set at 1000 °C for 1 h. Once the sintering was complete, the specimens were analyzed for various characteristics after they reached room temperature. Table 1 shows all of the mixture proportions used in this research.

For the control group (C100), 100% of clay was used. Unprocessed ISA (RS) (particle size < 4.75 mm) was used to replace 30 and 50% of clay to make RS30RC70 and RS50RC50 specimens for the first analysis, respectively. In the second analysis, we used processed materials (ground and sieved) (particle size < 0.15 mm) to replace 30 and 50% of clay to make S30C70 and S50C50 specimens, respectively. For the third analysis, 10, 20, 30, 40, and 50% of clay were replaced by WG, while the other 50% of clay was still replaced by processed materials similar to the second analysis to determine the effects on the quality of brick when using waste glass as flux.

### 2.3. Testing Procedure

In this study, the characteristic analysis included the compressive strength, water absorption rate, fire shrinkage coefficient, and weight loss on ignition. The test method for compressive strength refers to the [17] National Standards of the Republic of China (CNS). CNS 382 is specific about the specification of common bricks.

The area (*A*) of the compressed surface was obtained by measuring the length and width of the specimen. Due to the uneven surface of the brick, a piece of thick paper was placed on both the top and bottom of the brick to more evenly distribute the applied compression on the brick. We increased the compressive stress by 0.5~1 MPa per second until the specimen was damaged, at which point we recorded the maximum weight (*W*) applied (HT-9501 Hydraulic Servo Universal Testing Machine. Hung Ta instrument GO.LTD, Taiwan). We obtained the compressive strength by substituting the value of area (*A*) and weight (*W*) into Equation (1):

(1) $$C=\frac{W}{A}$$

where *C* is the compressive strength (MPa), *W* is the maximum weight (N) applied, and *A* is the area of the compressed surface (mm^2^).

Brick water absorption was determined by the method according to regulation CNS 382. After finishing the sintering process in the combustion chamber, we let the specimen cool to room temperature. The weight of the brick (*m*_1_) was obtained by using an electronic scale (FX-3000GD.A&D COMPANY, LIMITED, Tokyo, Japan). The brick was then placed in clean water at 25 °C for 24 h. After 24 h, we removed the extra water remaining on the surface of the brick with a damp cloth. The saturated surface-dry weight (*m*_2_) was then obtained by again using the electronic scale again. According to Equation (2), we obtained the water absorption ratio of the brick as follows:

(2) $$\;\mathrm{absorption}\;=\frac{m_{2}-m_{1}}{m_{1}}\times 100\%$$

where $m_{1}\;\mathrm{is}\;\mathrm{the}\;\mathrm{dry}\;\mathrm{weight}\;\left(g\right)$ and $m_{2}$ is the saturated surface-dry weight (g).

The adobe bricks formed after compaction were air-dried for 48 h using an electronic fan and then placed in an oven at 70 °C for 24 h. After all the drying processes, the weight of the adobe brick (*m_dry_*) was obtained using the electronic scale. Then, the bricks were placed into the combustion chamber for sintering, after which the bricks were cooled to room temperature and again weighed (*m_sin_*). We obtained the weight loss on ignition using Equation (3):

(3) $$\mathrm{weight}\;\mathrm{loss}\;\mathrm{on}\;\mathrm{ignition}=\frac{m_{dry}-m_{sin}}{m_{sin}}\times 100\%$$

where $m_{dry}$ is the weight of dried brick (g) and $m_{sin}\;\mathrm{is}\;\mathrm{the}\;\mathrm{weight}\;\mathrm{of}\;\sin\mathrm{tered}\;\mathrm{brick}$ (g).

The brick firing shrinkage test refers to [18]. ASTM C67-14 [19] specifically is about standard test methods for sampling and testing brick and structural clay tile. We obtained the volume of the adobe brick by measuring the length, width, and height of the dry brick (*V_dry_*). After the sintering process, we obtained the sintered volume (*V_sin_*) using the same method. The brick firing shrinkage ratio was then obtained according to Equation (4):

(4) $$\mathrm{firing}\;\mathrm{shrinkage}=\frac{V_{dry}-V_{sin}}{V_{sin}}\times 100$$

where $V_{dry}$ is the volume of the dry adobe brick (cm^3^) and $V_{sin}$ is the volume of the sintered brick (cm^3^).

First, we measured the weight of the sieved ISA before drying (*W*_1_). Then, we measured it again after it was dried in an oven at 105 °C for 24 h (*W*_2_). The oven-dried ISA was then put into the combustion chamber at 800 °C for 3 h. After the combustion chamber, the ISA was measured again after cooling down to room temperature (*W*_3_).

(5) $$\mathrm{water}\;\mathrm{content}=\frac{W_{1}-W_{2}}{W_{1}}\times 100$$

(6) $$\mathrm{ash}\;\mathrm{content}=\frac{W_{3}}{W_{1}}\times 100$$

(7) combustibility=100%−water content (%)−ash content (%)

where $W_{1}$ is the weight of the undried ISA, W_2_ is the weight of the dried ISA, and *W*_3_ is the weight of combusted ISA.

## 3. Results and Discussion

### 3.1. Characterization of Raw Materials Used for Brick Manufacturing

To understand the relationship between the characteristics of raw materials and sintering behaviors, we conducted XRD and XRF analysis on the ISA, WG, and clay used in the bricks. The chemical composition and basic properties of each material are shown in Table 2.

**Figure 2:**
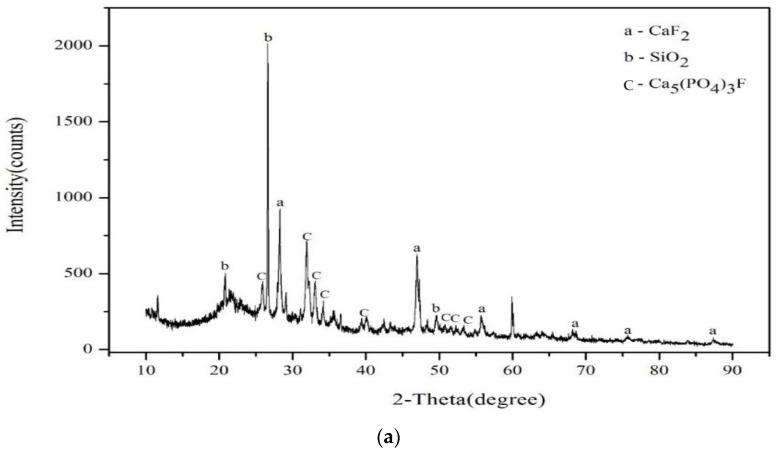

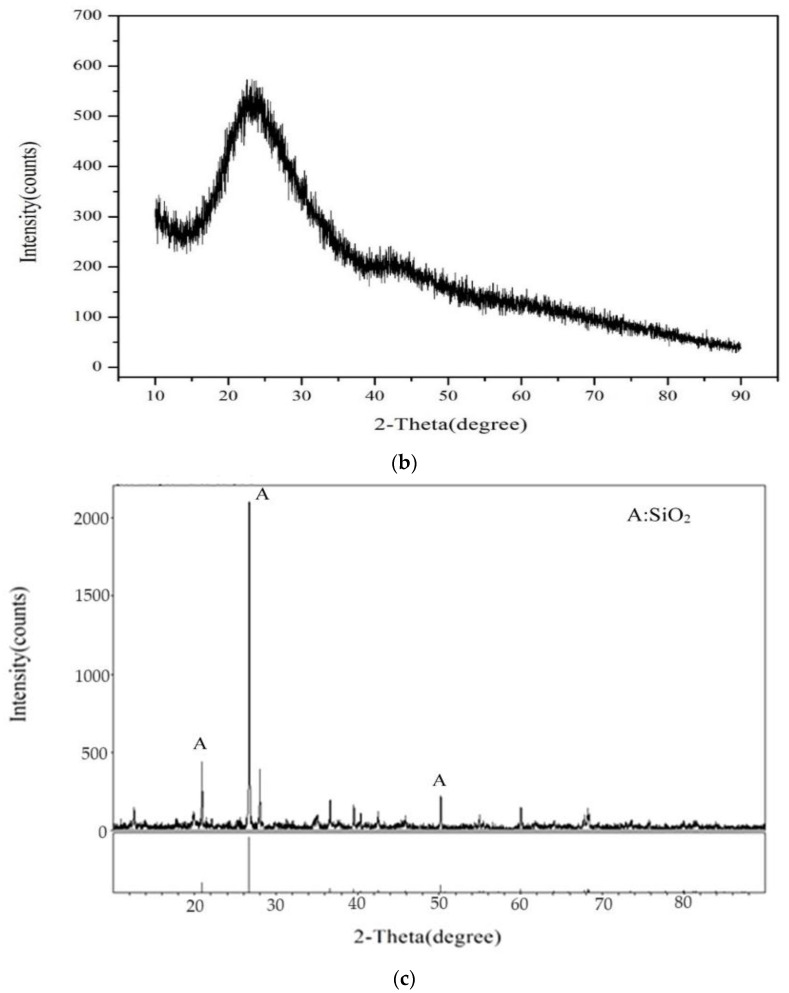
XRD data: (**a**) ISA; (**b**) WG; (**c**) clay.

According to XRF analysis, ISA contains up to 31.31% of calcium due to the manufacturing process. The content of silicon, aluminum, and ferrum were similar to that of clay and WG, but only that of silicon was too low for use as a sintering material. Skrifvars et al. (1994) [20] investigated the sintering mechanism of solid particles using coal-burning ashes from five different sources. The result indicates that silicon can easily form liquid viscous flow from 800 to 900 °C, which enhances the effect of viscous flow in sintering mechanics. At 985 °C, 79.7% of the melted mass was SiO_2_, which indicates that SiO_2_ is easily liquefied within this temperature range. Thus, the viscous flow would be the major sintering mechanism if the material contained a high level of silicon. The composition of clay, from which bricks are commonly manufactured, is mainly SiO_2_, Al_2_O_3_, Fe_2_O_3_, and incorporated water. Clay consists of 50~70% SiO_2._ Figure 2 shows that the silicon within ISA is mainly in a crystalline form of SiO_2_, according to XRD, which is similar to clay. However, the material in glass manufacturing would be melted first and then rapidly cooled, causing the molecules to not be in crystalline form. Thus, there would be no significant peaks shown on the XRD spectrum.

### 3.2. Effects of Industrial Sludge Ash Content on Brick

**Figure 3:**
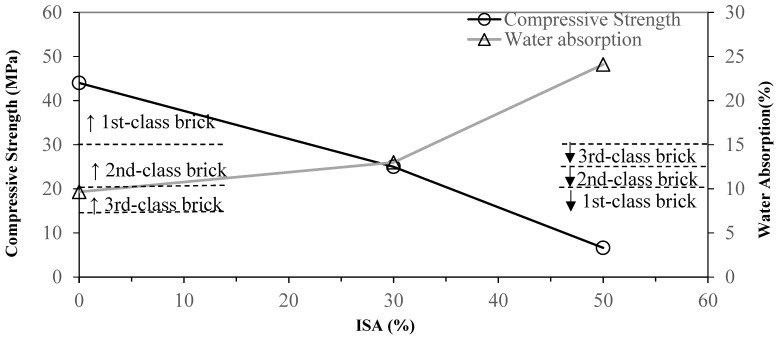
Effect of different unprocessed ISA contents on the water absorption and compressive strength of specimens.

According to the CNS 382 building brick specification, bricks are separated into first-, second-, and third-class bricks. Their compressive strengths exceed 30, 20, and 15 MPa, respectively, and their water absorption ratios are under 10, 13, and 15%, respectively. The comparison between replacing 30% and 50% of clay with unprocessed ISA in brick making and 100% clay brick is shown in Figure 3. The compressive strength of all clay brick is 44 MPa, which is therefore first-class brick. Replacing 30% of clay with unprocessed ISA decreases the compressive strength to the second-class brick standard. Replacing 50% of clay with unprocessed ISA drops the compressive strength to below the criterion of third-class brick.

The water absorption ratio of all clay brick made according to the method in our study reached the first-class brick standard. Replacing 30% of clay with unprocessed ISA led to a water absorption ratio matching that of the second-class brick standard, and replacing 50% of clay with unprocessed ISA made the water absorption ratio 24.1%, which failed to pass the lowest standard.

### 3.3. Process Improvement

**Figure 4:**
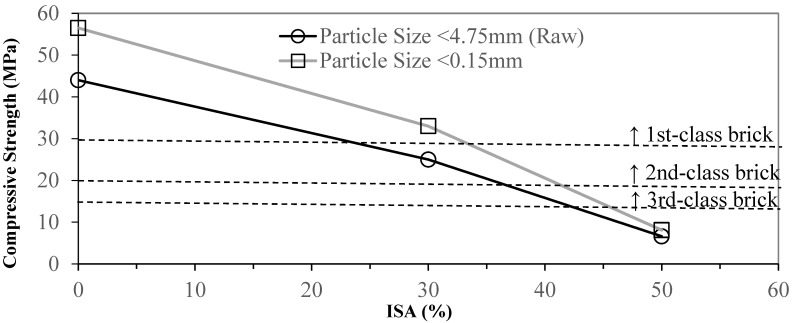
Effect of sieved ISA with different particle sizes on the compressive strength of specimens.

**Figure 5:**
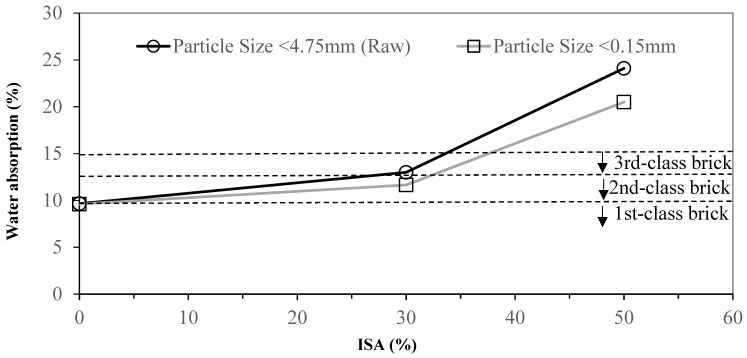
Effect of sieved ISA with different particle sizes on the water absorption of specimens.

**Figure 6:**
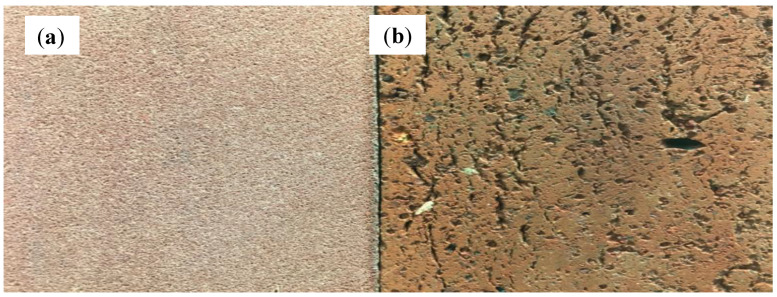
Cross-section images of bricks: (**a**) S30C70; (**b**) RS30RC70.

The compressive strength and water absorption ratio rapidly deteriorate as the particle size is increased. Therefore, we investigated how the particle size affects the quality of the bricks. We sieved the clay and ISA using a #100 screen (<0.15 mm). Applying the same method from the previous experiment, we replaced 30 and 50% of clay with fine ISA material. The results are shown in Figure 4 and Figure 5. The compressive strength was increased by 28% while using 100% of refined clay. The compressive strength was increased by 32% with 30% of refined ISA replacement. The compressive strength failed to meet any standard with 50% of refined ISA replacement. But the compressive strength was increased by 22% compared to using unprocessed ISA as replacement. The water absorption ratio was slightly more significant. Using 30% and 50% of refined ISA replacement, the water absorption ratio decreased by about 1.5% and 3.6%, respectively. Figure 6 shows that the cross-section of brick using the screened material was smoother and denser and with no obvious holes. The reduction in the particle size enhanced the sintering result, resulting in faster densification. The variety of particle sizes also affected the result. A wide variety of particle sizes might increase the density of the adobe bricks, as well as the distribution of the hole sizes. Therefore, the reduction in particle sizes causes a low sintering density. Thus, selecting a narrow range of particle sizes increases the sintering density.

### 3.4. Effects of Fluxing Agents on Properties of Brick

**Figure 7:**
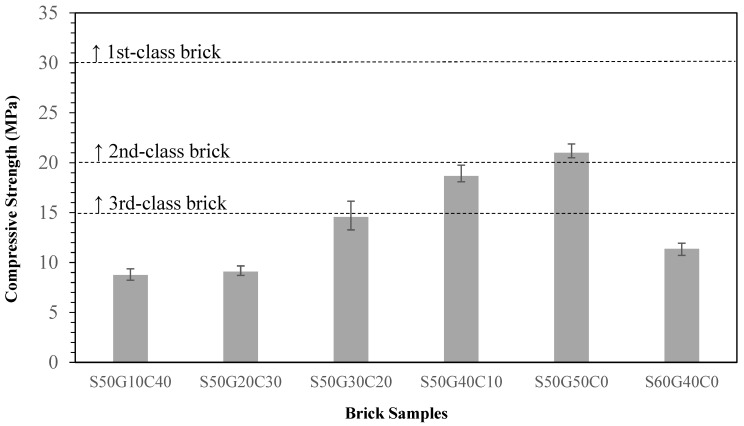
Effect of different WG contents on the compressive strength of specimens.

According to Figure 7, while adding 10~20% of WG did not affect the compressive strength much, which was about 8~9 MPa, adding 30% of WG significantly increased this strength up to 14~15 MPa. Parts of the specimens met the third-class criterion. While the amount of WG added increased between 40% and ≈50%, the compressive strength increased. We obtained the best compressive strength when we completely replaced all clay with WG. The strength attained the second-class brick standard of 20 MPa. However, if the ratio of the ISA added passed 50% to 60%, the compressive strength dropped to 11 MPa, which is similar to the case of adding 10~20% of WG.

**Figure 8:**
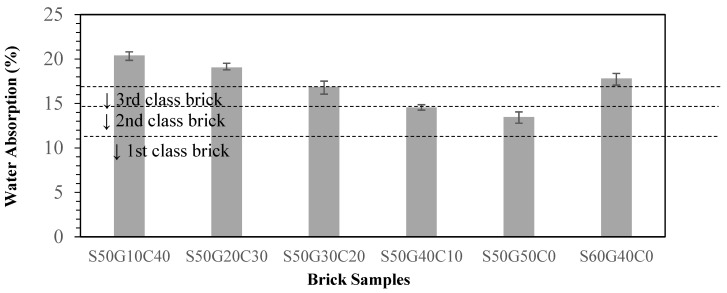
Effect of different WG contents on water absorption of specimens.

As WG does not have a fixed melting temperature, unlike crystalline materials, the atomic permutation of glass is more similar to that of a liquid (short-range order) [21] than a crystal (long-range order). Therefore, adding glass makes the specimens melt easier than the crystalline clay. Under the high-temperature sintering process, glass molecules are melted and densified together with the clay molecules, filling the gap between the sintered objects and enhancing the compressive strength of the bricks [22]. The increase in compressive strength should indicate a decrease in water absorption. As shown in Figure 8, the water absorption ratio decreased as more WG was added. When the WG replacement was 50%, the water absorption ratio met the second-class brick standard. However, the water absorption of specimen S60G40 (60% ISA) increased to 18% instead, as with the reverse situation of compressive strength.

**Figure 9:**
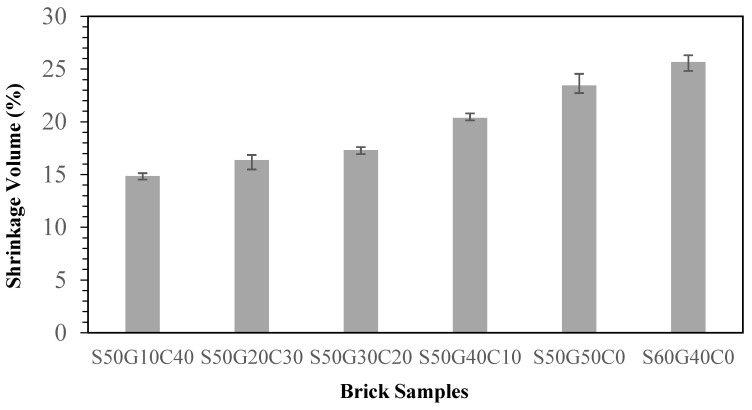
Effect of different WG contents on volume shrinkage of the specimens.

WG had a significant effect on the volume (Figure 9). With 10~50% of clay replaced by WG, the brick firing shrinkage increased from 15 to 23%. Specimen S60G40, however, did not exhibit the reverse situation as with compressive strength and water absorption ratio. The firing shrinkage still increased. This means that the firing shrinkage was affected by some substances within ISA besides the WG. The study by Park (2003) [23] indicated that adding 10% of CaO to sewage sludge will decrease the melting point. With the involvement of CaO, the crystalline phase of anorthite and diopside increases, and its crystalline phase enhances the hardness of the ceramic glass. Reich (2003) [24] indicated that there will be a substitution reaction between heavy metal and CaO. Adding different amounts of CaO will form different metal compounds, leading to different melting properties. Due to the bonds within crystal lattices, the melting point decreases. According to this research that indicates that CaO is considered as flux, we suggest that the CaO facilitates the WG to reach the melting phase. However, there is no solid proof of its effects on firing shrinkage.

**Figure 10:**
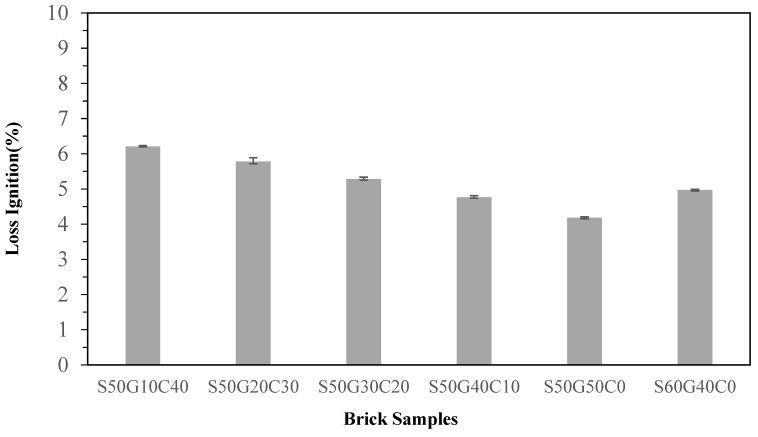
Effect of different WG contents in specimens on the loss of ignition.

The weight loss on ignition means the weight difference before and after sintering. The amount of loss depends on the evaporation of water, decomposition of organic compounds, disintegration and evaporation of inorganic salts, and minor heavy metal escape under high temperature. The effects of adding WG are shown in Figure 10. It was clearly affected by the contents of ISA and clay. WG is an inorganic salt, meaning that the weight loss on ignition mainly depends on the contents of ISA and clay. Specimen S50G50 had the most glass among all specimens. Therefore, its loss on ignition was the least at approximately 4.2%. Normally, if the specimens contain a high concentration of organic compounds or inorganic salts that decompose easily under high temperature, the loss of those components will create air holes following decomposition. The air holes compromise the inner structure of the specimens, affecting the characteristics such as the compressive strength and water absorption ratio. This result indicates that weight loss on ignition does relate to the compressive strength and water absorption ratio.

## 4. Conclusions

This study merely shows a feasibility of using ISA and WG as replacement of clay in the brick manufacturing industry. More application can be tested in, for instance, concrete making, road pavement, etc. The presented results show that replacing different amounts of clay with WG and ISA affect the quality of bricks. In addition, pre-processing the raw materials dramatically increases the outcome quality. Important conclusions of this research are listed below.

1. Grinding and sieving (<0.15 mm) the materials reduce the particle sizes and homogenize the materials, which increases the reacting area. This enhances the quality of the bricks. Compared to the pure clay standard, adding 30% of ground and sieved ISA increases the compressive strength by 31.6% and decreases the water absorption ratio by 11.8%
2. Adding WG increases the overall quality of the brick and the substitution potential. Adding 40~50% of WG meets the third-class brick standard.
3. Replacing 50% of clay with ISA while the other 50% of clay is replaced with glass is the best solution, with excellent waste utilization and the best compressive strength and water absorption ratio. However, considering the actual manufacturing process and the strength requirement, an ISA50%/WG40%/Clay10% ratio is recommended.

## Figures and Tables

**Table 1:** The mixture proportions of each brick sample. WG—waste glass; ISA—industrial sludge waste.

Brick Samples	ISA (%)		WG (%)		Clay (%)	
	Particle Size					
	<4.75 mm (Raw)	<0.15 mm	<4.75 mm (Raw)	<0.15 mm	<4.75 mm (Raw)	<0.15 mm
C100	-	-	-	-	100	-
RS30RC70	30	-	-	-	70	-
RS50RC50	50	-	-	-	50	-
S30C70	-	30	-	-	-	70
S50C50	-	50	-	-	-	50
S50G10C40	-	50	-	10	-	40
S50G20C30	-	50	-	20	-	30
S50G30C20	-	50	-	30	-	20
S50G40C10	-	50	-	40	-	10
S50G50C0	-	50	-	50	-	-
S60G40C0	-	60	-	60	-	-

**Table 2:** Chemical composition and other properties of the raw materials.

Properties	ISA	WG	Clay
SiO_2_ (%)	41.80	63.91	60.72
Al_2_O_3_ (%)	8.69	16.44	20.55
Fe_2_O_3_ (%)	7.67	0.29	9.62
CaO (%)	31.31	9.57	0.84
P_2_O_5_ (%)	13.58	-	-
SO_3_ (%)	3.58	-	-
ZnO (%)	0.37	0.23	-
Cr_2_O_3_ (%)	0.15	-	-
NiO (%)	0.13	-	-
K_2_O (%)	0.97	-	5.29
MnO_2_ (%)	-	-	0.16
CuO (%)	0.38	-	-
TiO_2_ (%)	0.34	0.17	1.34
Specific gravity	2.28	2.51	2.47
Water content (%)	1.64	-	1.89
Absorption (%)	23.54	-	11.35
Combustibility (%)	3.24	-	5.38

## Data Availability

Data are contained within the article.
